# Supplementary material for: Towards unified AI-driven fracture mechanics: the extended deep energy method (XDEM)
Source: Nat Commun. 2026 Aug 15;17:8492. doi: 10.1038/s41467-026-76748-1 (PMC13484471; doi:10.1038/s41467-026-76748-1)
Supplement: Supplementary file 3 [file 41467_2026_76748_MOESM3_ESM.zip › pic/Results/Crack_propagation/inclusion/crack_propagation.pdf]

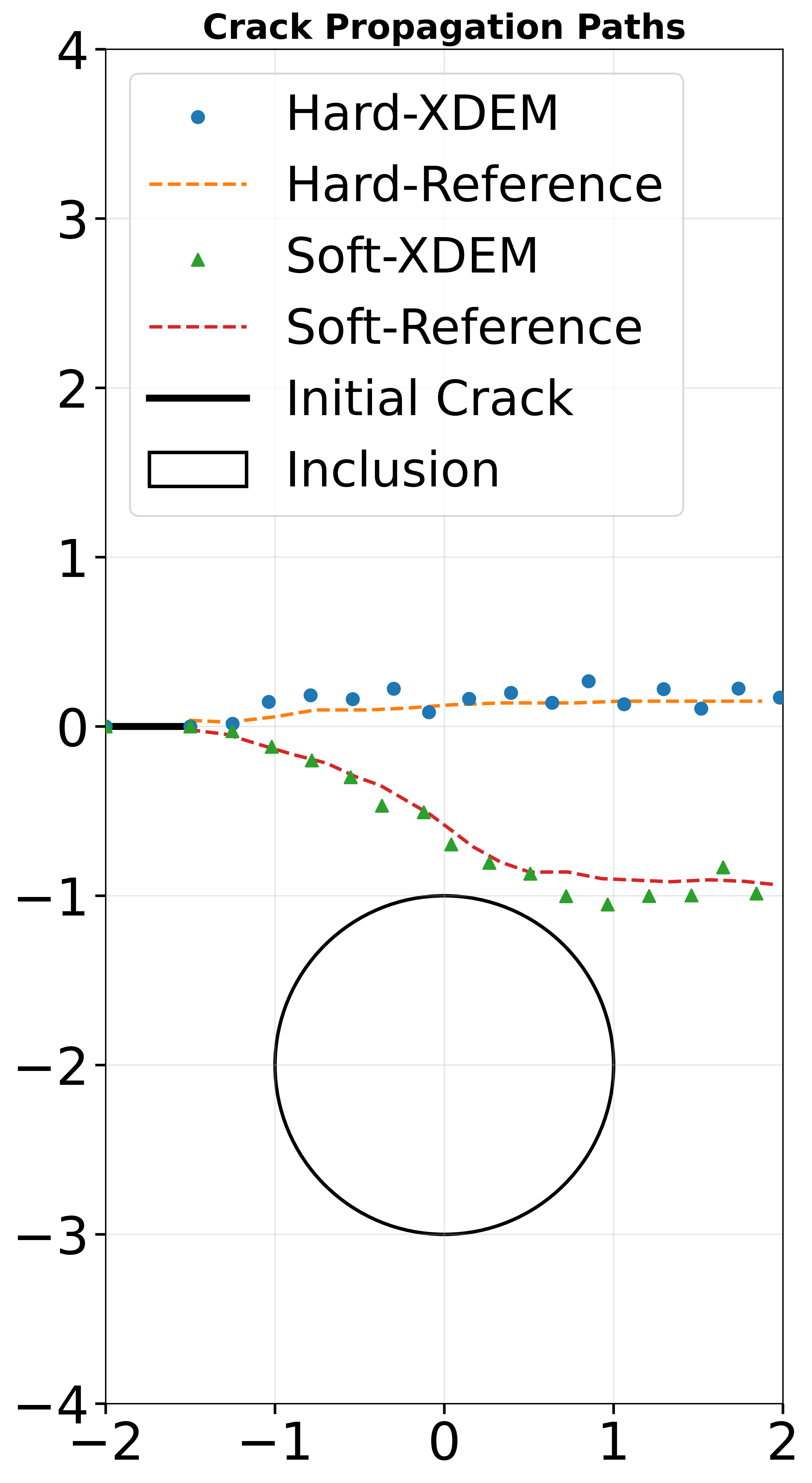

Crack Propagation Paths
Hard-XDEM
Hard-Reference
Soft-XDEM
Soft-Reference
Initial Crack
Inclusion
4
3
2
1
0
−1
−2
−3
−4
−2
−1
0
1
2
